# Red nodule on the lateral trunk

**DOI:** 10.1016/j.jdcr.2024.11.038

**Published:** 2024-12-24

**Authors:** Kerem Balan, Kübra Cicek, Aysen Karaduman, Ozay Gokoz

**Affiliations:** a Department of Dermatology and Venereology, Hacettepe University, School of Medicine, Ankara, Turkey; b Department of Pathology, Hacettepe University, School of Medicine, Ankara, Turkey

**Keywords:** eccrine porocarcinoma, skin neoplasms

An 81-year-old male patient presented with erythematous nodule on his axilla that has been present for approximately 6 months. Patient has widespread lesions on his body consistent with seborrheic keratosis (Fig 1, *A*). Polymorphic vessels and occasional erosions with erythematous and pink-whitish background were observed on dermoscopic examination (Fig 1, *B*). Histopathological examination revealed a dermal mass of epithelial proliferation of round to oval cells producing nodules with central necrosis. On high power, neoplasm exhibited tumor giant cells, pleomorphism, and frequent mitosis. Immunohistochemically the neoplastic cells were focally carcinoembryonic antigen (CEA) positive (Fig 2, *A* and *B*).

**Fig 1:**
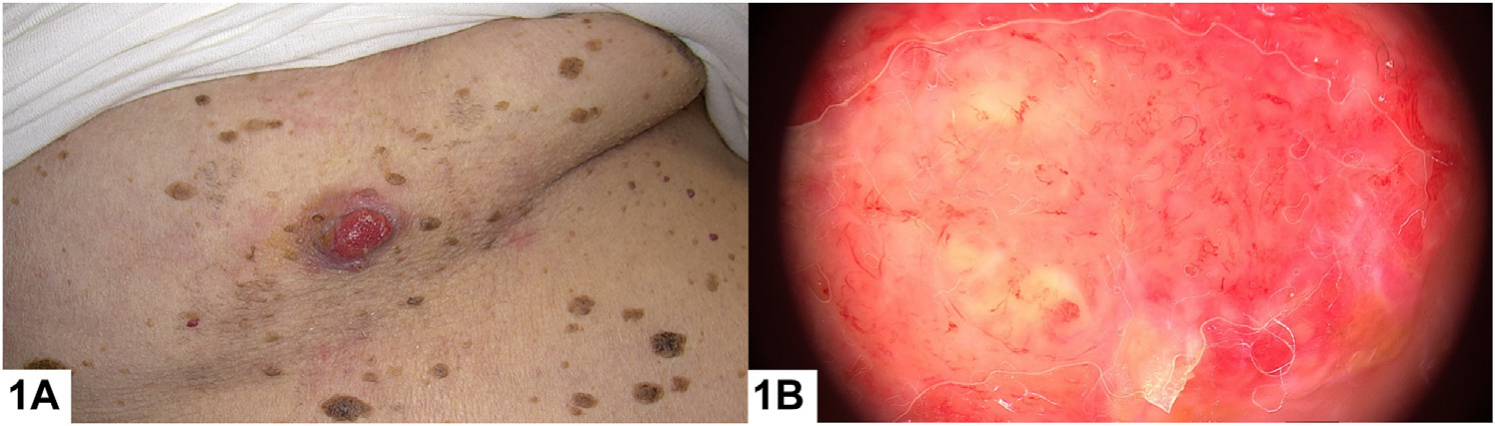

**Fig 2:**
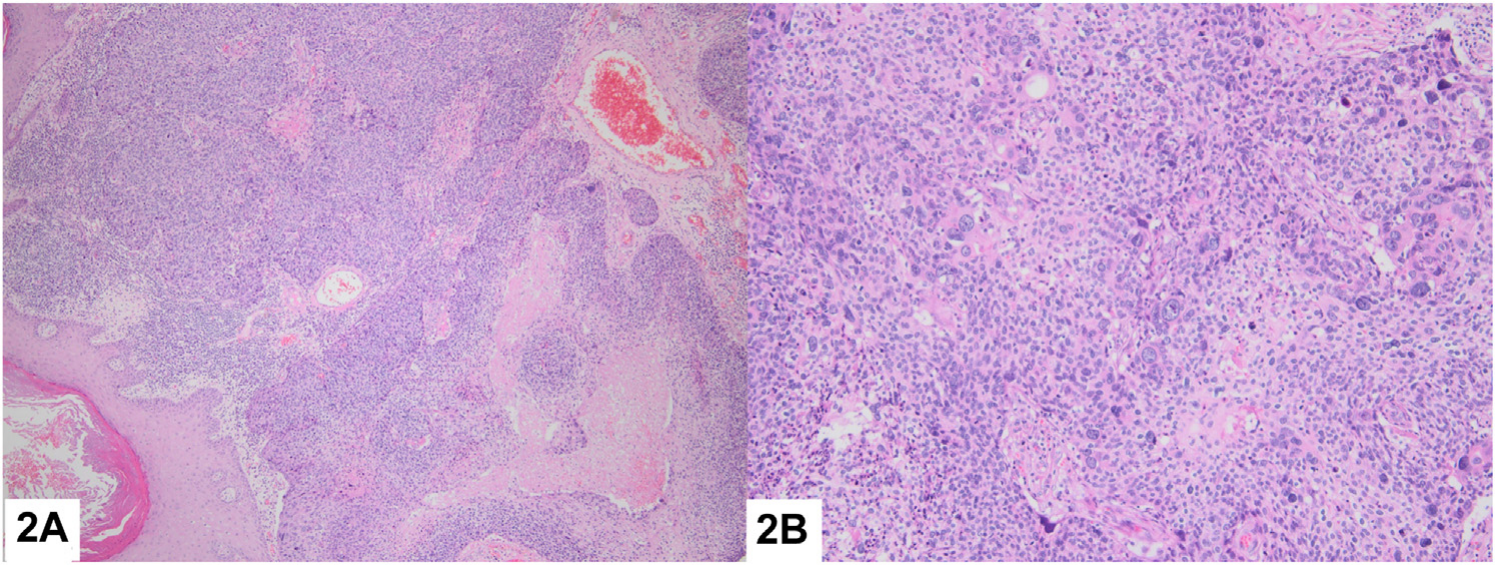

**Question 1: What is the most likely diagnosis?**

A. Eccrine porocarcinoma (EPC)
B. Merkel cell carcinoma (MCC)
C. Pyogenic granuloma
D. Amelanotic melanoma
E. Squamous cell carcinoma (SCC)

**Answer:**

A. Eccrine porocarcinoma (EPC) – Correct. EPC is characterized by large poromatous basaloid epithelial cells with ductal differentiation and cytologic atypia. This cytologic pleomorphism, along with increased mitotic activity, tumor necrosis, and an infiltrative growth pattern, aids in distinguishing EPC from benign eccrine poroma. EPC cells may also demonstrate squamous cell, clear cell, or spindle cell differentiation.^1^
B. Merkel cell carcinoma (MCC) – Incorrect. MCC is characterized by basaloid cells with a fine granular ‘salt and pepper’ chromatin pattern, prominent nuclear pleomorphism, and high mitotic activity, forming irregular nodules in the dermis and often exhibiting deep dermal and subcutaneous infiltration. Immunohistochemically, MCC typically shows positivity for CK20, Merkel cell antigen, synaptophysin, chromogranin, and CD56. MCC typically shows negativity for CEA.^2^
C. Pyogenic granuloma – Incorrect. Pyogenic granuloma is characterized by a rapidly growing, hyperplastic vascular lesion with numerous capillaries, and a mixed inflammatory infiltrate, often exhibiting a lobular pattern and surface ulceration.^3^
D. Amelanotic melanoma – Incorrect. Amelanotic melanoma is characterized by an invasive malignant tumor with atypical melanocytes that lack pigment, often presenting as poorly differentiated, infiltrative growth with significant pleomorphism and high mitotic activity.^4^
E. Squamous cell carcinoma (SCC) – Incorrect. Cutaneous SCC is primarily composed of keratinized cells, and it presents with keratin pearls and intercellular bridges. SCC can be distinguished from EPC by the presence of keratinization, epidermal connections, and more pronounced keratinization and desmoplasia. CEA positivity supports the diagnosis of eccrine differentiation.^5^

**Question 2: Which of the following is an immunohistochemical stain expected to be positive in EPC?**

A. CK20
B. MART1 (Melan-A)
C. S100
D. CEA, CD117, and cytokeratin-19
E. Smooth muscle actin

**Answer:**

A. CK20 – Incorrect. EPC generally does not show positivity for CK20, which is more commonly associated with MCC and certain gastrointestinal tumors.
B. MART1 (Melan-A) – Incorrect. This marker, associated with melanocytic differentiation, is generally negative in EPC.
C. S100 – Incorrect. This marker is usually negative in EPC, as it is more associated with melanocytic lesions and peripheral nerve sheath tumors.
D. CEA, CD117, and cytokeratin-19 – Correct. EPC often shows positive immunoreactivity for CEA and epithelial membrane antigen, further supporting its eccrine origin. In some cases, EPC may express p63 and p40, markers associated with squamous differentiation, which is observed in certain subtypes of EPC. Additionally, cytokeratin-19 and c-kit positivity has been identified in EPC, which can be useful in differentiating EPC from SCC.
E. Smooth muscle actin – Incorrect. This marker is usually negative in EPC, as it is associated with smooth muscle differentiation and not with eccrine tumors.

**Question 3: Which of the following is not associated with a poor prognosis in EPC?**

A. Pushing subtype
B. Lymphovascular invasion
C. More than 14 mitoses per high power field
D. Deep infiltration greater than 7 mm
E. Distant metastasis

**Answer:**

A. Pushing subtype – Correct. ECP has 3 histopathological variants: pushing subtype, infiltrative subtype, and pagetoid subtype. Among these variants, the pushing subtype is associated with a better prognosis compared to the other 2 subtypes.
B. Lymphovascular invasion – Incorrect. Lymphovascular invasion is associated with a poor prognosis.
C. More than 14 mitoses per high power field – Incorrect. This is associated with a poor prognosis.
D. Deep infiltration greater than 7 mm – Incorrect. This is associated with a poor prognosis.
E. Distant metastasis – Incorrect. This is associated with a poor prognosis.

## Conflicts of interest

None declared.
